# Supplementary material for: A Critical View of the Application of the APEX Software (Aqueous Photochemistry of Environmentally-Occurring Xenobiotics) to Predict Photoreaction Kinetics in Surface Freshwaters
Source: Molecules. 2019 Dec 18;25(1):9. doi: 10.3390/molecules25010009 (PMC7017383; doi:10.3390/molecules25010009)
Supplement: Supplementary file 1 [file molecules-25-00009-s001.zip › 3.2.4_gcc-4.4.0.zip!/3.2.4_gcc-4.4.0/3.2.4_gcc-4.4.0/doc/octave/html/interpreter/Sets.html]

Sets - Untitled


Next: Polynomial Manipulations,
Previous: Statistics,
Up: Top


---

## 26 Sets

Octave has a limited number of functions for managing sets of data, where a
set is defined as a collection of unique elements. In Octave a set is
represented as a vector of numbers.

— Function File: **unique** (x)  
— Function File: **unique** (x, "rows")  
— Function File: **unique** (..., "first")  
— Function File: **unique** (..., "last")  
— Function File: [y, i, j] = **unique** (...)  
> Return the unique elements of x, sorted in ascending order.
> If x is a row vector, return a row vector, but if x
> is a column vector or a matrix return a column vector.
>
> If the optional argument `"rows"` is supplied, return the unique
> rows of x, sorted in ascending order.
>
> If requested, return index vectors i and j such that
> `x(i)==y` and `y(j)==x`.
>
> Additionally, one of `"first"` or `"last"` may be given as
> an argument. If `"last"` is specified, return the highest
> possible indices in i, otherwise, if `"first"` is
> specified, return the lowest. The default is `"last"`.
>
>
>
> **See also:** union, intersect, setdiff, setxor, ismember.

- Set Operations
